# Where do you stand?: an exploration of perspectives toward feet, foot health, and footwear using innovative digital methods

**DOI:** 10.1186/s13047-023-00621-3

**Published:** 2023-04-28

**Authors:** Sue Skidmore, Yeliz Prior, Christopher Nester, Sam Bird, Cristina Vasilica

**Affiliations:** 1 School of Health & Society, University of Salford, Brian Blatchford Building, Frederick Road Campus, Salford, M6 6PU UK; 2 MacKay Building School of Health and Rehabilitation, Keele University, Keele, ST5 5BG UK

**Keywords:** Feet, Foot health, Beliefs, Attitudes, Prevention

## Abstract

**Background:**

The cost of losing foot health is significant to the person, healthcare systems, and economy, with diabetes related foot health issues alone costing over £1 billion annually in the UK. Yet many foot health problems are preventable through alternative health behaviour. It is therefore important to understand how feet, foot health and footwear are conceptualised to gain understanding about how these might influence foot health behaviour and inform health messages that seek to protect or improve foot health through altered health behaviour. This research seeks to explore attitudes and beliefs and identify phenomena that may act as barriers or motivators to the proactive self-management of foot health.

**Methods:**

Public conversations involving 2,699 expressions related to feet, footwear or foot health on Facebook, Twitter, and Instagram were extracted. Conversations on Facebook and Twitter were scraped with NVivo’s NCapture plugin whereby data is extracted and downloaded to NVivo. Extracted files were uploaded to the Big Content Machine (software developed at the University of Salford) which facilitated the search for keywords ‘foot’, ‘feet’, ‘footwear’, ‘shoe’, and ‘shoes’. Instagram was scraped by hand. Data was analysed using a Thematic Analysis approach.

**Results:**

Three themes were identified; 1) connections and disconnections derived from social and cultural constructs, 2) phenomena beyond attitudes and beliefs that relate to symbolic representations and the impact when foot health is lost, and 3) phenomena relating to Social Media as a conduit for the exploration of attitudes and beliefs.

**Conclusions:**

This novel research exemplifies complex and sometimes incongruous perspectives about feet including their value for what they facilitate, contrasted with negative feelings about the negative impact that can have aesthetically when feet work hard. Sometimes feet were devalued, with expressions of disgust, disconnection, and ridicule. The importance of contextual, social, and cultural phenomena with implications for optimising foot health messages. Knowledge gaps including factors related to children’s foot health and development, and how to treat foot health problems. The power of communities with shared experience to influence decisions, theories, and behaviour about foot health was also revealed. While people do talk about feet in some social contexts, it is not always in a way that promotes overt, positive foot health behaviour. Finally, this research demonstrates the benefit of exploring perspectives in uncontrived settings and illuminates the potential utility of social media (SoMe) platforms Facebook, Instagram, and Twitter as vehicles to promote foot health self-management behaviour that is responsive to the social and demographic variances of engagers who inhabit those spaces.

**Supplementary Information:**

The online version contains supplementary material available at 10.1186/s13047-023-00621-3.

## Background

The need to engage in positive foot health behaviour is significant, where the burden of impaired foot health associated with co-morbidities such as diabetes is high [1], with diabetes related foot health issues such as ulceration and amputation costing approximately £1 billion annually in the UK alone [2]. Need for care will out-pace the sustainability of the healthcare system [3]. Many foot health problems are preventable through alternative health behaviour, for example evidence shows that where self-management is practiced it can reduce foot disability scores [4]. The impact when foot health is lost includes diminished quality of life [5] risk of falls [6] impaired mental health [7, 8], diminished social, and physical well-being [8], capacity to work, and relationships with others [9]. When foot health is compromised to the extent that it places the foot at increased risk of amputation, for example following diabetic foot ulceration [10], this leads to mortality rates comparable to cancer [11].

Observable judgments in the form of attitudes [12] and the beliefs that inform them [13] are understood to have a direct impact upon health-related behaviour [14], and successfully changing health behaviour can be difficult [15]. Therefore, understanding attitudes and beliefs is a central tenet of overcoming barriers to behaviour change [16]. Attitudes and beliefs about feet, footwear [17], and foot health [18] are understood to be influenced by constructs outside of health-related parameters for example where an emotional attachment to shoes is expressed [17], and how feet are sometimes associated with disgust [19]. However, attitudes and beliefs are also malleable [20]. Therefore, understanding attitudes and beliefs that have the capacity to influence motivation to engage proactively with foot health is central to informing practice level strategies and addressing the associated significant healthcare burden, where prevention is preferable to cure [21]. This would be consistent with contemporaneous policy that places health ownership on the individual, encouraging self-management [22–24].

Evidence about the prevention of foot problems is often situated in domains where foot health is already at increased risk, and not about establishing proactive self-management practices that would encourage maintained foot health in a generally healthy population. Therefore, research need not limit the identification of attitudes and beliefs to a specifically defined clinical cohort such as those with a chronic illness or occupation that places the foot at risk. Instead, it is important to illuminate phenomenon that may be important to inform a true person-centred approach that acknowledges the importance of respecting factors specific to the individual that might influence care [25]. This includes understanding attitudes and beliefs toward feet, foot health, and footwear of a wider population, and not just those whose foot health is at increased risk. Therefore the central aim of this research is to explore attitudes and beliefs toward feet, foot health, and footwear, to illuminate phenomena that has the potential to impact upon motivation to engage with proactive foot health behaviour.

Understanding this, one approach to investigating attitudes and beliefs is to use heterogenous data sources to ensure any exploration embraces a wide range of perspectives. SoMe has been shown to meet this criterion, providing accessible, ubiquitous, and authentic data [26] for example, utilising Twitter as a ‘window’ for the expression of attitudes and beliefs [27]. Data scraping is a digital research method that lends itself to understanding cultural phenomenon [28], lived experience [28], behaviour [29], attitudes and beliefs [30], self-expression, self-documentation, information sharing, and social interaction [31]. It provides an opportunity to efficiently explore concepts [32], from ‘big data’, across a heterogenous sample where up to 95% of digital content is an unstructured [33] sharing of private thoughts in public domains [34]. This provides a unique platform through which to meet our aim to explore perspectives toward foot health and identify phenomena that may act as barriers or motivators to proactively self-managing foot health.

Facebook (Meta, California), Twitter (Twitter inc., San Francisco), and Instagram (Meta, California) are the three most popular social networks accessed in the UK [35] where unstructured text communication is central. Exploring the 3 platforms affords a spread across demographic groups such as different socio-economic groups and age ranges for example where Facebook engagers tend to be older [36] than those of Instagram, where 95% of those aged 18–24 are active on SoMe [37]. However, while only 13% of those aged 50–64 use Instagram, 50% of those aged 65 + and over 70% of those aged 50–64 used Facebook [38]. This cross-platform approach lends itself to diversity [39], and improves inclusivity [40]. Finally, by utilising data that has been observed in a natural social space, this removes the potential for an interviewer/participant dynamic that can bias the results during interviews [41].

## Methods

Digital methods is a novel approach and as such there are several terms of reference that may not be familiar. Clarification of these terms is provided in Table 1. Digital methods was used in other health contexts to identify health needs [42], demonstrate impact [43] or the role of health influencers on Youtube [44]. Building on the approaches above, this study combines different digital tools and analytical approaches to expand knowledge in the field of foot health.

**Table 1:** Clarification of terms

Terms	Explanation
SoMe	Shortened version of social media
Tweets	Character limited expressions posted on Twitter (280 characters)
Posts	Expressions and messages posted in online social media environments
Data scraping	A digital method that involves the extraction of large datasets in a format that facilitates analysis
Big data	Heterogeneous, large, and complex datasets, that derive from many sources that gives opportunity for the exploration of phenomenon
Unstructured text	Freeform text that does not have a predefined format, examples include e-mails, presentations, word documents
NCapture	Web-browser extension that enables web content to be gathered and imported into NVivo (qualitative data analysis software), as PDF files
Big Content Machine	Bespoke software developed at the University of Salford that facilitates searches for keywords from big data sets

### Ethics

Ethical approval was gained from the University of Salford (HSR 1819–106). Ethical principles relating to data scraping outlined by the Association of Internet Researchers were adopted. Gaining informed consent when utilising ‘big data’ is not always feasible or meaningful if data is cleaned of personally identifiable information [45], where it is aggregated such that no comments or quotes are directly reproduced [46], and where data has been collected from multiple and diverse platforms.

General Data protection Regulation (GDPR) regulations highlight that where research activity is both in the public interest and conducted by legitimate research agents [47], research occupies a position of privilege, if the process safeguards anonymity [34]. These principles were applied to the data scraping methods of this research. To mitigate the risks and ensure full anonymity of data and participants, this research does not include individual quotations (posts, comments, tweets, or any type of data).

### Initial search framework

An iterative and exploratory hand search of Facebook, Twitter, and Instagram was undertaken to establish the utility of this research method and embellish the search strategy that was informed by a prior literature review and clinical experience. Additional information is provided in relation to the search sectors (Additional information 1) and search terms (Additional information 2). Both ‘@’ and ‘#’ prefixes were searched on Instagram and Twitter due to the potential to render search results.

### Platform and search process

Data from Facebook and Twitter were scraped utilising the NVivo NCapture web extension to download data from specified Facebook pages and Twitter search results. A manual search and extraction were performed on Instagram. Following a manual exploration of all 3 platforms, several interest areas where expressions were evident about feet, foot health and footwear were identified (see Additional file 1). Each platform has its own microenvironment and therefore the search terms for each platform differed. Details of platform specific search terms is provided in Additional file 2.

The extracted dataset was imported into excel spreadsheets for qualitative analysis. The data was searched for keywords ‘feet’, ‘foot’, ‘footwear’, ‘shoe’, and ‘shoes utilising the Big Content Machine (BCM), a bespoke data search tool developed at the University of Salford that facilitates keyword searches within large datasets. The number of expressions from Facebook and Twitter is provided in Table 2. The first 20 Instagram stories were identified and analysed by hand for expressions including the keywords.

**Table 2:** Expressions from each identified sector across the 3 platforms

Sector	Facebook	Twitter	Instagram
Running	72	38	12
Swimming	129	0	7
Gym/fitness	6	2	20
Pilates/yoga	3	3	33
Gardening	0	0	19
Dancing	5	2	11
Walking/rambling	17	0	21
Pathology	255	46	69
Beauty and aesthetics	9	6	20
Wedding footwear	4	6	21
Footwear/shoes	4	132	13
Ageing/caring	77	10	26
Parenting/pregnancy	432	27	75
Feet general	230	60	6
Popular press/lifestyle	741	13	17
**TOTALS**	**1984**	**345**	**370**

A second researcher (CV) critically analysed the results with the first researcher. Thematic analysis [40] was then utilised to systematically make sense of the data and ensure that the unstructured textual data was methodically analysed.

### Data analysis

**Fig. 1:**
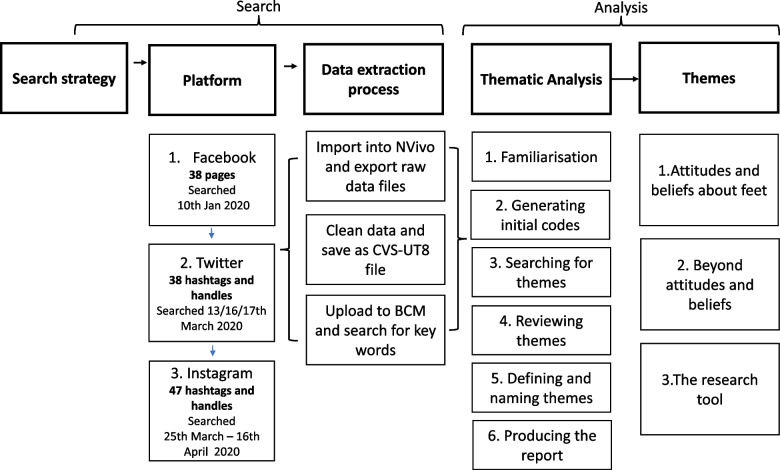
Digital methods extraction and analysis process: a diagrammatic representation of the sequencing and steps undertaken to extract and analyse the data from Facebook, Twitter, and Instagram

The sequence and process of data extraction undertaken by the first author and analysis across the 3 platforms is shown in Fig. 1. The Facebook analysis from phases 1–3 was utilised to inform analysis of Twitter and Instagram, before the final analysis phases [4–6] were sequentially employed to inform the inter and intra-platform analysis. Some common phrases were retained as exemplars to bring the aggregated data to life, no direct quotes were utilised. The data was coded descriptively to summarise the data [48], utilising an inductive approach, to condense the raw data [49].

The 6 stages of Thematic Analysis (Table 3) was used to iteratively analyse the data across the 3 platforms.

**Table 3:** Six stages of Thematic Analysis [50]

Phase		Description of the process
1	Familiarisation	Transcribing data, reading, re-reading the data, noting initial ideas
2	Generating initial codes	Coding interesting features systematically across the data set, collating data relevant to each code
3	Searching for themes	Collating codes and gathering data into potential themes
4	Reviewing themes	Checking if the themes work in relation to the codes, and generating a thematic map
5	Defining and naming themes	Ongoing refinement of themes, generating clear names for each theme
6	Producing the report	Final analysis results into a comprehensive report of the analysis

Credibility, dependability, transferability, and confirmability, are often regarded as important tenets of qualitative research, where each of these criteria can be demonstrated through various mechanisms [51]. How this research meets each of these criteria as described by Shenton [51] is detailed in Table 4 below.

**Table 4:** Credibility, dependability, transferability, and confirmability

**Credibility**	Involvement of a large number of ‘informants’, peer scrutiny by a second researcher, and frequent debriefing sessions with PhD supervisors
**Dependability**	Reporting of the research design and operational detail of data gathering
**Transferability**	Transparency about the boundaries of the research such that the extent to which the results are transferable are clear (limitations, data collection methods and sources, number of data collection incidences, time period)
**Confirmability**	Provision of an audit trail (description of the platform and search process above) and the utilisation of a large dataset with many ‘informants’ to ensure that the results reflect the data and not researcher bias

## Results

While there was some cross-platform commonality, there were several key differences in relation to the tone and nature of how platforms were utilised and engaged with, and these are identified throughout the results, but the final theme is dedicated to identifying platform specific phenomenon. Table 5 gives an overview of the themes, sub-themes, and a brief description in relation to the phenomenon identified.

**Table 5:** Themes, sub-themes, and a brief description

Theme title	Sub-themes	Description
**1.** **Attitudes and beliefs about feet**: *connect and disconnect*	**Wider social and cultural phenomenon**: foot bones aren’t just connected to your heel bone	The ways in which we are socially and culturally connected by concepts related to feet, and conversely, disconnections exemplified by negative language, foot health knowledge gap, and a disconnect between footwear and foot health
	**when feet stand apart**: disconnect and disassociation	
**2.** **Beyond attitudes and beliefs**: *symbolic representations and impact when foot health is lost*	**Symbolic references**	These sub-themes inform understanding of perceptions toward feet, foot health behaviour, the value (or not) of feet, and impact when foot health is at risk
	**The value of feet, and the impact when foot health is lost**	
**3. The research tool**: *platforms and how users engage*	**Platform specific overview**	There were areas of divergence and convergence both within and across the 3 platforms related to SoMe architecture, engagement norms, and lifeworld’s of those engaged within it
	**The power of SoMe to connect and inform**	

### Theme 1: attitudes and beliefs toward feet; connect and disconnect

Beliefs and attitudes toward feet, foot health, and footwear were expressed in a myriad of ways, demonstrating the utility of SoMe as a data collection mechanism for foot health related phenomena. These results demonstrate how foot health in itself is not always consciously valued specifically in relation to the foot, but for what good foot health facilitates and how footwear is sometimes a competing influence in relation to that foot health. These factors have implications for motivation to self-care and seek treatment for foot health.

#### Sub-theme 1: wider social and cultural phenomenon—foot bones aren’t just connected to your heel bone

Expressions about feet were more often about what feet facilitate such as dancing, travel, hobbies, social occasions, and sport. Positive attitudes were expressed for feet that will not ‘let you down’, fast running feet, or special occasions such as getting married. Beliefs related to how feet are hardworking, while concurrently longing for pretty feet. Connecting within social worlds and a sense of self was consistently evidenced, for example footwear and self-expression, getting comfortable with new trends such as wearing trainers with dresses, and fitting in socially and at work, even if it induced pain or pathology. Excitement toward activity shoes was expressed but mostly where it prioritised aesthetics of the footwear, above function and performance.When foot health had been lost, connections were established through engagement with others that shared a similar foot health experience rather than with the podiatrists present on SoMe who were proactive in demonstrating capacity to treat that condition.

Positive attitudes about feet were less prevalent, where the vast majority related to babies and young children who were celebrated as being comfortable with their feet, exploring them and being barefoot. This conversely suggests that adults often are not comfortable with their own feet which has implications for proactivity to manage foot health. there were several common expressions that related to this, indicating how feet *should* be hidden away and not visible to others. Beliefs connected to commonly held theories about caring for feet were also expressed, but were often contrary to foot health focussed clinical evidence. These include the role of devices for non-pathological flat feet, theories about ‘over’ pronation, advocates for barefoot shoes and forefoot running, running technique, and the impact on muscles, joints, or callus formation, and how shoes are coffins or traps that lock feet in.. Several expressions connected the season to a change in attitude or behaviour, such as getting feet summer ready.

#### Sub-theme 2 – when feet stand apart: disconnect and disassociation

There was a predominance of negative attitudes about feet themselves, suggesting stigma around feet and fetish, ridicule, and mockery in relation to feet, and a strong association with those common pathologies that were associated with stigma. Table 6 provides some examples.The most prominent example relates to a foot health Facebook page that is ‘about’ sharing foot health problems and advice, where some posts broke those rules of engagement with expressions that ridiculed feet. Even in activity areas where foot health is important, feet were not valued, for example, describing other swimmers as ‘foot touchers’ as a derisory comment toward other swimmers. Comments about foot fetish were often utilised to humiliate, linked to disgust, or common pathologies such as fungal feet and bunions, and qualified with expression such as ‘lmao’^1^ and ‘lol’.^2^

**Table 6:** Negative descriptors relating to feet

Foot related negative descriptors and expressions to describe feet			
Disgusting	Manky plates of meat	cheesy	Gross
Repulsive	Webbed	Big	Creepy
Hobbit feet	Ugly	Dusty	Dirty
Smelly/sweaty	Strange	Monkey toes	Elephant feet
Putrid	Stinky	Nutty Professor feet	Hate feet

Disconnect was expressed toward health and service providers, such as commercially led foot measuring services offering inconsistent service or advice, and healthcare professionals who could not explain or help manage symptoms, reinforced by expressions of how only those with a shared experience can truly understand. There was also disparity over whether the benefit was worth the risk when treatments were painful such as steroid injections, and knowledge gaps e.g., the importance of checking feet for people with diabetes, and whether first shoes hindered development or helped support ambulation. There was also confusion about footwear e.g., when flat shoes caused pathology, or avoiding high heels did not prevent it. There was also frustration toward institutional footwear policies that did not prioritise comfort, foot health, or practicality, such as schools and workplaces. Several posts also suggested a disconnect with the lower limb, with implications for propensity to engage proactively with lower limb health, with links to a lack of intellect or competence. Examples include references to a person having brains in their feet, being unable to tie shoelaces, footballers having nothing upstairs and interviewing a person’s feet. Shame was also suggested when foot pathology caused deformity, for example some Instagram posts suggested strategies to turn back time through cosmetic treatments, to hide pathology.

### Theme 2: beyond attitudes and beliefs: symbolic representations and the impact when foot health is lost

While not beliefs and attitudes, these sub-themes inform understanding of perceptions toward feet, and concepts that may influence foot health behaviour through the expression of symbolic references, and the impact when foot health is compromised.

#### Sub-theme 1: symbolic references

Physical representations such as tan lines, dirt, and callus were expressed as a metaphorical badge of honour, where feet were valued for how they facilitated some activities but where the result was impaired foot health. This suggests that the impact of doing many things that people enjoy and value has a negative impact upon foot health.Similarly footwear was not always linked to foot health and protection, where footwear was utilised as an expression of self, with shoes sometimes customised to reflect self-identity and personality. There were symbolic negative attitudes expressed between feet and worthiness, for example being fit (or not) to polish somebody’s shoes and kissing the feet of revered statues. The importance of footwear was also indicated when it was no longer wearable due to pathology or post-pregnancy, symbolising something lost.

#### Sub-theme 2: the value of feet, and the impact when foot health is lost

The impact of diminished foot health on activities of daily living such as going to the bathroom, gardening, walking, sports, and shopping, with implications for social participation or capacity to care for others. One Tweet acknowledged a Catch-22 scenario where a diabetic foot ulcer meant that healthcare professional recommended activity to prevent disease progression could no longer be continued. Being on feet all day, and activities such as sightseeing and running were also impeded by foot pain or pathology. Several posts related to the impact of pregnancy, and how for many foot pain, hobbling, or swelling was the only negative experience, how foot shape changes, and can impact footwear choice. The language tended to be emotive or relate to a sense of futility such as likening living with diabetic foot pathology to being on a boat full of holes (Table 7).

**Table 7:** Language used to describe the impact when foot health is lost

Emotive language to describe impact		
Excruciating	Fear	Feet in boiling water
Indescribable	Hot	Hell
Agony	Icy	Nightmare
Constant	Suffering	Depressed
Pain	Swelling	Deformity
Puffy ankles	Soreness	Aching
numbness	Scarring	Worst pain ever
Feeling suicidal	Anxiety	Overwhelmed

Many posts shared the impact impaired foot health can have on mental health. Fear was associated with both having a toenail surgically removed, and the prognosis when foot health in diabetes was ignored for 3 years, indicating fear as one reason for foot health reactivity. For many, aesthetics mattered when foot health was impaired, such as bunions that made feet look like ‘paddles’. Sequalae included impaired sleep, reduced quality of the next day, and sense of self for example when clinically appropriate footwear was indicated.

Some motivators to care for foot health were indicated when diminished foot health from pathology or pregnancy also impacted capacity to work, particularly if the role was non-sedentary. Barriers to proactive foot care self-management including financial implications related to the cost of footwear and treatment products or services. However very few posts related to seeking appropriate clinical treatment. Footwear could also create a health dichotomy when safety boots provided protection but were sometimes a barrier to returning to work after foot pathology. Others alluded to the positive impact of treatment with a professional foot health clinician or aesthetic treatment. These included multiple expressions about pedicure-related expressions suggesting ‘love’ for ‘new’ feet post-pedicure, and celebrations of ‘new’ feet post-surgery. These posts were infrequent and greatly outnumbered by posts sharing negative impact.

### Theme 3: SoMe specific engagement in relation to foot health

How people related to feet, foot health, and footwear differed across the three platforms, and this needs to be transparent such that implications for self-management to prevent foot health can be situated within the context from where the data was extracted.

#### Sub-theme 1: platform specific overview

There were several characteristics that were unique to each platform in relation to who engaged, how, and for what purpose Table 8).

**Table 8:** Overview of differentiating characteristics of the 3 SoMe platforms

Feature	Facebook	Twitter	Instagram
Engagement characteristics	• Pathology • Reaching out/advice • Tagging others	• Selling products	• Supportive, celebratory
Length	• Unlimited • Often long posts	• Limited to 280 characters	• Unlimited/ sometimes just an image • Variation in length
Engagers	• Individuals	• Professionals and individuals	• Individuals and professionals
Other characteristics	• Organised into pages with specific interest areas • More emotive	• Neutral/less emotive	• All posts accompanied an image • Abstract concepts • Comparatively positive

On Facebook, bi-directional engagement with others sharing an experience and trusting the advice was prominent both in and outside of pathology, with disconnect from those that did not, which included clinicians. This included expressions that ‘rated’ the advice from others with a shared experience over that of foot health specialists.

Many Tweets were orchestrated by professionals, but even though the topics posted reflected the common pathologies often stigmatised, or conditions where there was a lack of knowledge or that had a significant impact, there was still little engagement. In relation to foot health, there were lots of products and ‘quick fixes’ on offer to meet foot health needs though these were commercially derived and not necessarily underpinned by evidence. The potential for SoMe to influence purchasing habits was identified, with tweets relating to trends and where to find them, and advice on how to emulate a look, primarily targeted at women.

What this research illuminates that directly relates to the medium utilised for data extraction is that what matters to people is not always foot health itself, but what was transparent across all 3 platforms was the importance and value of feet for what they facilitate, the importance of aesthetics, and the lack of knowledge about how to achieve a healthy looking foot that also supports that activity foot pain free. This is important to informing truly person-centred care to meet these gaps and provide the care that holistically meets needs and resonates such that foot health is more proactively engaged with.

#### Sub-theme 2: the power of SoMe to connect and inform

Several Facebook page posts indicated the power of SoMe to connect people with shared experiences. While some self-management strategies appeared to be underpinned by efficacious clinical advice, others were clearly attributed to advice from companies selling products. An example includes alleviating neuropathy with a homemade tea tree oil foot spray, or equipment to self-treat an ingrowing toenail. On Twitter and Instagram health education was promoted, such as exercise or checking feet daily. Twitter was also about connecting service and product providers to the marketplace. Empowerment and foot health was occasionally advocated outside of a pathological domain but related to a specific activity, for example exercises to strengthen dancer’s feet. However, one Facebook post shared information from a clinical encounter, that was taken to a subsequent appointment for another engager, positively informing one clinician’s practice. There was an implicit trust in some expressions predominantly from those who had shared a similar experience, relating to foot pathology diagnoses, treatment or product recommendations, or non-clinicians such as the reflexologist who informed an engager that serious foot pathology was the result of a traumatic event in childhood, signifying the gap between efficacious clinical practice and the wider public’s perception of that evidence and scope of professional practice.

## Discussion

This study explores expressions extracted from SoMe that relate to feet, foot health and footwear using a novel digital methods approach, and offers the opportunity to illuminate perspectives about feet, footwear, and foot health in naturally occurring social spaces. The digital approach described differs from traditional methods in foot health research, with particular novelty in the fact that data is undisturbed by any dynamic between the researcher and the researched [52]. Also, cross-platform differences were explored, which validated the use of extracting data from 3 platforms with different demographic representation and engagement dynamics. This is consistent with the findings of Davidson [53], who alludes to a tendency to ‘shape shift’ depending on the social situation.

Overwhelmingly, expressions about feet were negative. Constructs such as foot fetish, ‘foot touching’, referencing feet to humiliate others, and a negative connection between feet and common pathologies, suggests that foot positive expressions about feet may be perceived as socially deviant. This is supported by evidence that relates feet with disgust [19] and footwear fetish [54]. This was exploited in the utilisation of #footfetish as a mechanism to engage social media users, often where the posts or tweets did not relate to fetish. This is important because stigma and social influences are known to be powerful blockers to positive behaviour change [55]. Therefore, the impact of the wider social context is a powerful influencer of the beliefs, attitudes, and associations that drive behaviour. It follows that where these perspectives and the social worlds that influence them are embedded in negative associations this may have a negative impact upon willingness to adopt proactive foot self-care behaviour to prevent or address foot health problems. This has important implications for influencing positive behaviour change in the future [56].

This is reinforced by expressions that indicated an above and below the waist divide, where what is below is often expressed as less valued. This resonates with the opinion piece by Ingold [57], who references Marxist philosophy, suggesting that bipedal humans are divided at the waist, while feet propel a person *within* the natural world, what is above the waist is related to intelligent designs that have the capacity to act *upon* it.

Many expressions related to the visible appearance of feet, for example signs of pathology or the importance of footwear to self-expression and fitting in social worlds or specific environments such as work. While feet were valued for what they facilitate, as ‘badges of honour’ that visibly displayed the effects of effort, this was contrasted with the negative impact upon feet both aesthetically and physically. The importance of aesthetics is also acknowledged in relation to coveted footwear, reinforcing evidence that signifies shoes as status symbols [58], and power expression [59], and the impact on confidence when footwear choice is removed due to pathology [60, 61]. Further, expressions about babies’ feet were unanimously positive, contrasted with predominantly negative expressions about the appearance of the ageing foot, suggesting the relevance of aesthetics in relation to feet.

Importantly, many aspects of belief systems evidenced in our data are not founded in a current evidence-base. There was confusion about how best to care for feet such as when to put a child in a first pair of shoes, ‘normal’ childhood foot development, how to treat common pathologies, and caring for the foot at risk. Mistrust also featured in relation to commercial entities such as children’s footwear providers, inconsistent foot measuring and the practical utility of the advice given by some foot health professionals. This demonstrates an unmet need and opportunity to promote good habits, and behaviour underpinned by clinical evidence, from a young age, by engaging new parents in positive behaviour. This substantiates existing research that found a lack of empowerment amongst parents and carers [62]. Further, while there were acknowledgements of a desire to care for aching or hardworking feet this was mostly addressed by recommendations to visit a pedicurist and not a foot health professional.

This was also a feature once foot health was compromised or at risk, when the value of good foot health became apparent. This research reflected the powerful influence between those with a shared experience, and a lack of trust toward those that had not. This manifested in expressions of confidence in recommendations for treatment modalities, products, and services, and an absence of signposting to clinical diagnosis and treatment.

Further research based on the themes identified here should explore these concepts in more depth including knowledge gaps, how to meet them, the importance of aesthetics and how these phenomena act most powerfully as a barrier or motivator to positive foot health behaviour. Gathering the perspective of the clinician might be a valuable complement to the outcomes here, to ensure that the implications for professional practice (and policy) achieve a true therapeutic alliance. Indeed, integrating the factors identified here into practice and professional training will accelerate adoption of a more person-centred approach, placing factors that are important to the individual central to professional priorities [63], that are underpinned by behaviour change theories that offer an evidence based frame of reference.

The findings from this study are consistent with engagement trends reported elsewhere, where Instagram posts tend to be positive, negativity and emotive posts are more prevalent on Facebook [39] and Twitter users are less likely to utilise the platform for social relationships [64]. This is important as it highlights the value and potential of utilising different social environments to explore attitudes and beliefs, and engage people, and the power of the social situation to influence what is expressed there. Expressions about fetish are an example of how one phenomenon was represented differently cross-platform. Significantly, Instagram offered insight into a predominantly younger demographic [65], with notable attitudinal changes for example toward the foot in pathology and attitudes toward new footwear trends. This suggests a utility for SoMe as a vehicle to promote foot health messages that is sensitive to the dynamics of the demographics, attitudes, beliefs, and behaviours of those that inhabit that space.

## Conclusion

This research illuminates a latent value of good foot health linked to what feet facilitate, and the importance of contextual, social, and cultural phenomena to expressed attitudes and beliefs in the presence of both good foot health and pathology. Yet most expressions about feet were negative. Where there was an investment in foot health, for example when health is diminished the trust that develops amongst people with a shared experience was powerful. It also demonstrates knowledge gaps such as factors affecting children’s foot health, how to access foot health professionals, and related scope of practice. Finally, people do talk about feet in some social contexts, but not always in a way that promotes positive foot health behaviour.

### Limitations

This data scrape was exploratory in nature. The interest areas and phenomenon that were explored was guided by a manual data scrape, literature review, researcher clinical and research experience, and the granularised data from existing studies that focused on specific scenarios such as a pathology-based cohort. Scraping data from SoMe has many advantages, however it also poses several limitations such as opacity around the algorithms that govern what data is returned from largescale extraction, and how some data types are prioritised over others. This is outside of the researchers’ control. While this approach assures anonymity, it also limits capacity to provide and account for demographic information which may have provided important insights as to the population of interest. Further, this exploration was defined by the chosen expressions and search terms within each platform, and many more expressions exist that were not identified or explored. This was further inhibited by the dynamic nature of SoMe, where data is added to all the time, yet this research was viewed through a lens that captured a single moment in time. This lens also only allowed for the capture of expressions from people who proactively engage in SoMe. Further, due to platform limitations Instagram had to be scraped by hand as the search software was not congruous to a machine facilitated Instagram search. Finally, although there were advantages to SoMe facilitating the exploration of qualitative unstructured text across a broad data set, this also eliminated any potential to dive deeper into the experiential phenomenon that was highlighted.

## Supplementary Information

Additional file 1. Activities and phenomena searched across the 3 platforms. Identifies the range of activities and phenomena where expressions about feet were found to be evident. Many other areas were reviewed in the exploration of all 3 platforms prior to the data scrape, for example football. In this example, no expressions in the manual search revealed an insight into perspectives, attitudes, or beliefs about feet, footwear, or foot health.Please check Additional files if captured correctly.Checked and captured correctly

Additional file 2. Table of search terms modified for each platform. Provides the search terms for each platform in relation to how the activities and phenomena in Additional file 1 were represented on each platform.

## Data Availability

The extracted and analysed datasets utilised in this study are available from the corresponding author upon reasonable request.

## Footnotes

1 Lmao is an abbreviation for ‘laughing my ass off’.

2 Lol is an abbreviation for ‘laugh out loud’.
