# Supplementary material for: Where do you stand?: an exploration of perspectives toward feet, foot health, and footwear using innovative digital methods
Source: J Foot Ankle Res. 2023 Apr 28;16:25. doi: 10.1186/s13047-023-00621-3 (PMC10141949; doi:10.1186/s13047-023-00621-3)
Supplement: Supplementary file 1 — Additional file 1. Activities and phenomena searched across the 3 platforms. Identifies the range of activities and phenomena where expressions about feet were found to be evident. Many other areas were reviewed in the exploration of all 3 platforms prior to the data scrape, for example football. In this example, no expressions in the manual search revealed an insight into perspectives, attitudes, or beliefs about feet, footwear, or foot health.Please check Additional files if captured correctly.Checked and captured correctly [file 13047_2023_621_MOESM1_ESM.docx]

## Additional file 1: Interest areas and phenomena identified across the 3 platforms

- Running
- Gym
- Fitness
- Swimming
- Pilates
- Yoga
- Gardening
- Dance
- Walking/rambling (top 5 of each pages returned)
- Arthritis
- Diabetes
- Heart health
- Mehndi feet
- Pedicure
- Wedding/wedding footwear/wedding shoes (top 3 of each search pages returned)
- Travel/adventure (top 5 of each pages returned)
- Ageing/carers (top 5 of each pages returned)
- Barefoot
- Parenting UK
- Pregnancy UK
- Feet/foot (top 5 of each pages returned)
- General lifestyle (top 10 papers, lifestyle sites identified through google searches)
