# Supplementary material for: Where do you stand?: an exploration of perspectives toward feet, foot health, and footwear using innovative digital methods
Source: J Foot Ankle Res. 2023 Apr 28;16:25. doi: 10.1186/s13047-023-00621-3 (PMC10141949; doi:10.1186/s13047-023-00621-3)
Supplement: Supplementary file 2 — Additional file 2. Table of search terms modified for each platform. Provides the search terms for each platform in relation to how the activities and phenomena in Additional file 1 were represented on each platform. [file 13047_2023_621_MOESM2_ESM.docx]

## Additional file 2: Table of search terms modified for each platform

| **Facebook search terms** | **Twitter hashtags/handles** | **Instagram hashtags/handles** |
| --- | --- | --- |
| Running | #footpain | Diabetesfeet |
| Swimming | #fitnessfeet | Arthritisfeet |
| Gym | #Gymfeet | Vascularfeet |
| Fitness | #Yogafeet | Swimmingfeet |
| Pilates | #Pilates | Fitnessfeet |
| Yoga | #Dancingfeet | Gymfeet |
| Gardening | #Walkingfeet | Pilatesfeet |
| Dance | #Barefoot | Dancefeet |
| Walking | #Running | Walkingfeet |
| Ramblers | #Activefeet | Travellingfeet |
| Rheumatoid arthritis | #Pregnancyfeet | Barefootfeet |
| Diabetes | #Childrensfeet | Runningfeet |
| Heart health | #Shoes | gardenersfeet |
| Mehndi feet | #Lovefeet | Activefeet |
| Pedicure | #Weddingshoes | Travellersfeet |
| Wedding | #Mehndi | pregnancyfeet |
| Wedding footwear | #Pedicure | childrensfeet |
| Wedding shoes | #Ageing | Mumsfeet |
| Footwear | @britishheartfoundation | Babiesfeet |
| Shoes | @VersusArthritis | Shoes |
| Travel | @@NRAS | Loveshoes |
| Adventure | @diabetes.co.uk | Lovefeet |
| Ageing | @loosewomen | Feet |
| Aging | @mumsnet | Weddingfeet |
| Carers | @carerstrust | mehndifeet |
| Barefoot | @diabetesUK | Pedicure |
| Parenting UK | @Healthline | Pedicurefeet |
| Pregnancy UK | @HuffPostParents | Ageingfeet |
| Feet | @HuffPostUK | Agingfeet |
| Foot | @NHS@SagaUK | Carersfeet |
| Daily Telegraph | @PregnancyOrg | Footpain |
| Guardian | @Guardian | M&S footwear collaborators |
| Metro | @LondonEveniningStandard | Loose Women |
| The Sun | @Independent | The Telegraph |
| Independent | @NationalGeographicTravel | Metro |
| Daily Express | @TheSun | The Sun |
| London Evening Standard | @The Times | The Independent |
| Times and Sunday Times | @WalkingBritain | The Daily Express |
|  |  | London Evening Standard |
|  |  | The Times & Sunday Times |
|  |  | Mumsnet |
|  |  | NRAS |
|  |  | Versus Arthritis |
|  |  | Diabetes UK |
|  |  | BHF |
|  |  | Healthline |
|  |  | Saga |
|  |  |  |
|  |  |  |
|  |  |  |
|  |  |  |
|  |  |  |
| **TOTAL: 38** | **TOTAL: 38** | **TOTAL: 47** |
